# The Impact of Regional Climate Change on Malaria Risk due to Greenhouse Forcing and Land-Use Changes in Tropical Africa

**DOI:** 10.1289/ehp.1103681

**Published:** 2011-09-07

**Authors:** Volker Ermert, Andreas H. Fink, Andrew P. Morse, Heiko Paeth

**Affiliations:** 1 Institute of Geophysics and Meteorology, University of Cologne, Cologne, Germany; 2 School of Environmental Sciences, University of Liverpool, Liverpool, United Kingdom; 3 Institute of Geography, University of Würzburg, Würzburg, Germany

**Keywords:** climate change, highland malaria, malaria, malaria model, malaria projection, Sahel

## Abstract

Background: Climate change will probably alter the spread and transmission intensity of malaria in Africa.

Objectives: In this study, we assessed potential changes in the malaria transmission via an integrated weather–disease model.

Methods: We simulated mosquito biting rates using the Liverpool Malaria Model (LMM). The input data for the LMM were bias-corrected temperature and precipitation data from the regional model (REMO) on a 0.5° latitude–longitude grid. A *Plasmodium falciparum* infection model expands the LMM simulations to incorporate information on the infection rate among children. Malaria projections were carried out with this integrated weather–disease model for 2001 to 2050 according to two climate scenarios that include the effect of anthropogenic land-use and land-cover changes on climate.

Results: Model-based estimates for the present climate (1960 to 2000) are consistent with observed data for the spread of malaria in Africa. In the model domain, the regions where malaria is epidemic are located in the Sahel as well as in various highland territories. A decreased spread of malaria over most parts of tropical Africa is projected because of simulated increased surface temperatures and a significant reduction in annual rainfall. However, the likelihood of malaria epidemics is projected to increase in the southern part of the Sahel. In most of East Africa, the intensity of malaria transmission is expected to increase. Projections indicate that highland areas that were formerly unsuitable for malaria will become epidemic, whereas in the lower-altitude regions of the East African highlands, epidemic risk will decrease.

Conclusions: We project that climate changes driven by greenhouse-gas and land-use changes will significantly affect the spread of malaria in tropical Africa well before 2050. The geographic distribution of areas where malaria is epidemic might have to be significantly altered in the coming decades.

The climate system of the earth has a strong affect on human life that may cause a wide range of health effects. Humans very likely affect the climate by greenhouse-gas emissions, which lead to anthropogenic global warming. Warm and humid atmospheric weather conditions, such as those prevailing in tropical lowlands throughout most of the year, make areas suitable for water-associated diseases like malaria (e.g., Githeko et al. 2000). Such diseases are therefore affected by climate changes associated with increased temperatures and altered precipitation (Martens et al. 1997). The Intergovernmental Panel on Climate Change (IPCC) expects that climate change will have a mixed effect on the spread of malaria (Confalonieri et al. 2007). In certain areas, disease distribution will probably contract; in other places, such as highlands, it will expand; and malaria seasonality might be significantly altered.

Many other factors can affect malaria, and some may counteract the effects of weather and climate (Confalonieri et al. 2007; Lafferty 2009). In the last decade, an increase in financing for malaria control (Snow et al. 2008) facilitated efforts to combat malaria (World Health Organization 2008), including the distribution of insecticide-treated nets (Noor et al. 2009). Recently, Gething et al. (2010) used evidence-based malaria maps to show that despite global warming during the 20th century, a global recession of malaria was observed. The currently observed malaria range, especially outside of Africa, is less widespread than expected based on current knowledge of climate suitability. Indirect effects of climate change on malaria might be more important than direct effects (Saugeon et al. 2009). Changes in agricultural productivity due to changes in climate could provoke migration and might lead to increased urbanization, which results in lower transmission rates (Hay et al. 2005; Keiser et al. 2004).

Climate and weather conditions become less important under the presence of human interventions, which play an important role in Africa (e.g., Craig et al. 2004). However, the decline in endemicity might also be caused by altered rainfall patterns. After the drought in the Sahel in the 1970s (Nicholson 2005), a decrease in malaria transmission was observed (e.g., Mouchet et al. 1996).

In general, the hypothesis that climate change and climate variability are responsible for observed changes in malaria remains controversial. Various researchers have argued that observed increases in malaria in the East African highlands were caused by increased temperatures (e.g., Alonso et al. 2011; Bonora et al. 2001; Loevinsohn 1994; Omumbo et al. 2011; Pascual et al. 2006), whereas others have concluded that the increase in malaria in the highlands was caused by other factors (e.g., Hay et al. 2002). Pascual et al. (2008) reported that disease and meteorological factors might complement each other and interact at different time scales. Therefore, the assessment of the potential change in malaria risk caused by climate change and climate variability remains an important topic.

Most studies that have assessed the future of malaria used output from coarse spatial resolution of general circulation models (GCMs), and some were limited to comparatively small regions (e.g., Ebi et al. 2005; Peterson 2009; Tanser et al. 2003; Thomas et al. 2004; van Lieshout et al. 2004). From a meteorological point of view, the direct use of output from coarse global climate models has several limitations. First, the grid resolution of GCMs (~ 200–400 km) is too coarse to adequately capture the effects of local terrain on temperatures and rainfall. Second, model biases were not corrected, and third, future changes in the land surface characteristics were hitherto almost never taken into account. From the malaria transmission point of view, simple and poorly validated models have often been used to analyze malaria transmission.

The present study used a novel and comprehensive approach for the malaria projection in sub-Saharan Africa. It used bias-corrected output from a high-resolution regional climate model (RCM), the regional model (REMO) that was driven by greenhouse-gas and land-use and land-cover (LUC) changes. We applied the REMO data to drive the Liverpool Malaria Model version of 2010 (LMM_2010_; see Ermert 2010; Ermert et al. 2011a, 2011b) to assess the annual entomological inoculation rate (EIR_a_; i.e., the number of infectious mosquito bites per human per year). Our study used the EIR_a_ values as input data for the *Plasmodium falciparum* infection model proposed by Smith et al. (2005) (the S_2005_ model) to produce reasonable asexual parasite ratios for children < 15 years of age (PR_<15_; i.e., the rate of children being infected with the malaria parasite). PR_<15_, expressed as a percentage, is widely measured by microscope or by the polymerase chain reaction that enables an estimation of the disease burden. The infection rate usually varies with age and is highest among children (e.g., Smith et al. 2005).

Both the inclusion of LUC changes into REMO and the integration of an RCM with two malaria models at a 0.5° latitude–longitude resolution are novel malaria-modeling approaches.

## Materials and Methods

*Regional model (REMO) climate scenarios.* The assessment of the impact of climate change on the disease of malaria is based on data from REMO, which is a limited-area model with a horizontal grid resolution of 0.5°. We produced an ensemble of three REMO integrations for each climate change experiment to take into account the internal variability of the model. Our study assessed the degree of uncertainty of future climate due to A1B and B1 greenhouse-gas emission scenarios (Nakićenović et al. 2000). The A1B scenario includes a rapid economic growth, the use of new and efficient technologies, and a balanced emphasis of energy sources. The B1 scenario is ecologically more optimistic. It is also characterized by a rapid economic growth with a reduction of material intensity and an introduction of clean and resource-efficient technologies.

The LUC changes for the African tropics are in line with a scenario from the Food and Agriculture Organization (2006). We prescribed the strongest LUC changes for the A1B scenario, and we used somewhat weaker changes under B1 (for details, see Paeth et al. 2009).

*Surface temperature changes.* Surface temperatures generally increase according to the REMO climate projections [see Supplemental Material, Figure 1 (http://dx.doi.org/10.1289/ehp.1103681)]. The strongest warming signal occurs under the A1B greenhouse-gas scenario paired with the strongest LUC changes. The projected surface warming is most pronounced at the end of the simulation period and is strongest in tropical Africa. Projections based on the B1 scenario with weaker LUC changes are similar, although temperature increases are generally 1°C lower than projected under A1B. In contrast with IPCC assessments (Christensen et al. 2007), the reduced vegetation cover in REMO causes a shift in the strongest temperature increase from arid areas to humid areas; here, reduced evapotranspiration causes higher surface temperatures (Paeth et al. 2009).

**Figure 1:**
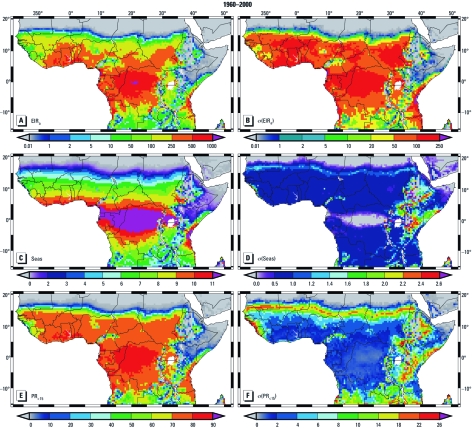
LMM_2010_ and S_2005_ model-simulated present-day (1960–2000) malaria distribution. (*A*–*D*) LMM_2010_-modeled EIR_a_ (*A*), σ(EIR_a_) (*B*), Seas (*C*), and σ(Seas) (*D*). (*E,* *F*) S_2005_ model estimates of PR_<15_ (*E*) and σ(PR_<15_) (*F*). The value scales for *A* and *B* represent the number of infectious bites per human per year, *C* and *D* are months, and *E* and *F* are percentages. All values that are not standard deviations are averaged values for the particular period.

*Rainfall changes.* REMO simulates a reasonable climatological pattern of annual rainfall in Africa (Paeth et al. 2009). Under the A1B and B1 scenarios, REMO projects a prominent decrease in rainfall in most parts of West and Central Africa and a positive rainfall trend on the windward side of the Guinean Mountains (~ 8°N, 12°W) and over the Horn of Africa [see Supplemental Material, Figure 2 (http://dx.doi.org/10.1289/ehp.1103681)]. In contrast, changes in rainfall are more irregular and less pronounced under the A1B scenario without induced LUC changes (Paeth et al. 2009; see their figure 6). Paeth et al. (2009) argued that the projected decrease in precipitation would result from reduced evapotranspiration of the diminished vegetation due to human activities. As a consequence, the continental water recycling is diminished.

**Figure 2:**
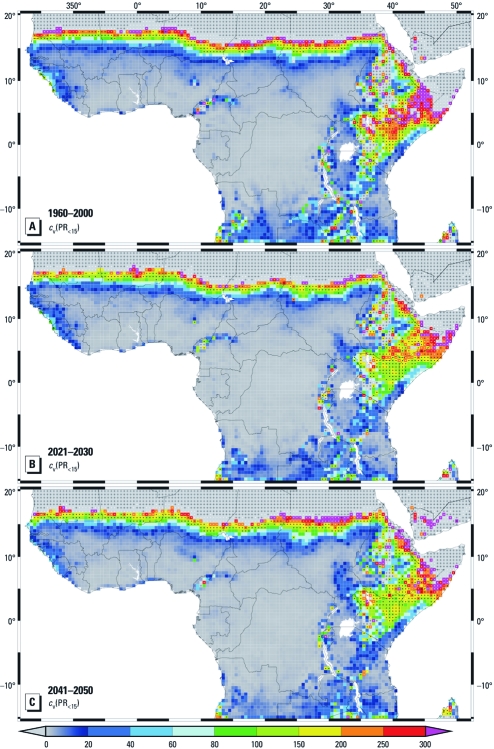
Risk assessment of malaria epidemics using the S_2005_ model: *c*_v_ of the asexual parasite ratio of children (PR_<15_) indicating epidemic risk for 1960–2000 (*A*), 2021–2030 (*B*), and 2041–2050 (*C*). For the future values, the climate model was driven by the A1B scenario. Black crosses mark grid boxes at which the LMM_2010_-simulated malaria transmission (EIR_a_ > 0.01 infective bites per human per year) occurs in < 5% of the years. White and black dots refer to a frequency of malaria transmission occurrence at each grid box of 5–50% and 50–95%, respectively. For boxes without crosses or dots, malaria transmission occurs for all the simulated years of the considered period. The value scale for *A*–*C* represents percentages.

Bias correction. Climate models are subject to biases that, when they are systematic in nature, can be compensated for by appropriate statistical methods. In our case, we corrected the simulated weather data to ensure realistic LMM_2010_ input data using climatological differences between the REMO data and observed monthly rainfall from the Climatic Research Unit (version CRU TS 1.1; University of East Anglia, Norwich, UK), as well as daily temperatures from ERA40 (European Centre for Medium-Range Weather Forecasts Re-analysis, 40 years; see Ermert 2010 for details).

*Malaria modeling.* The *P. falciparum* infection model. A nonlinear relationship exists between the entomological inoculation rate (EIR) and *P. falciparum* infection in children (Smith et al. 2005). Smith et al. (2005) fitted various mathematical functions to 119 published paired EIR_a_ and PR_<15_ observations from Africa (Hay et al. 2005) by the maximum likelihood method. The best-fitting model (the S_2005_ model) assumed heterogeneous infection rates and no immunity to reinfection but includes superinfection (i.e., an infection that follows a previous infection):


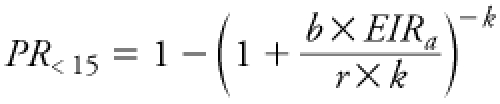
,

where *b* represents the transmission efficiency (the probability that an infectious mosquito bite causes infection) and 1/*r* is the expected time for parasite clearance. The S_2005_ model assumes heterogeneous infection rates following a Γ (gamma) distribution, with a mean of 1 and variance 1/*k*. The estimated parameters were *b*/*r* = 0.45, 1/*k* = 4.2. Note that the S_2005_ model reveals a large uncertainty because of the large variability of the observations (Smith et al. 2005, see their figure 1).

The Liverpool malaria model. The LMM is a weather-driven, mathematical–biological model of malaria that was originally formulated by Hoshen and Morse (2004) and was applied, for example, by Jones and Morse (2010), who validated it against a 20-year clinical record for Botswana. It simulates daily malaria transmission and infection rates based on daily mean temperature and 10-day accumulated precipitation. However, the model does not include some detailed aspects of the malaria infection, for example, the immune status of humans.

Recently, Ermert et al. (2011a, 2011b) constructed the improved LMM_2010_ to include structural changes. For example, they parameterized hydrological conditions by a fuzzy distribution model (Ermert et al. 2011a), which considers the flushing of breeding habitats by excessive precipitation. Their study calibrated and extensively validated the LMM_2010_ by numerous field observations from West Africa in terms of 11 entomological and parasitological variables (Ermert et al. 2011b). They measured, using a problem-adapted skill score, the ability of the model to capture the range of observations in the vicinity of weather stations. Their study provided evidence that the LMM_2010_ simulates realistic EIR_a_ values and reasonable malaria seasons. The results show that the model reproduces the strong observed interannual variability of EIR_a_. The performance is somewhat weaker regarding parasitological variables. For this reason, we applied the S_2005_ model for the simulation of *P. falciparum* infection rates. We passed the EIR_a_ values from the LMM_2010_ to the S_2005_ model to produce meaningful PR_<15_ values.

We used the LMM_2010_ for the simulation and projection of the spread of malaria under past and future weather conditions. Three LMM_2010_ runs were simulated on a 0.5° grid by daily temperatures and rainfall amounts from different REMO integrations of the present-day climate of 1960–2000. Subsequently, we carried out malaria projections for 2001–2050, which are based on the two different REMO climate projections. We then ran the S_2005_ model integrations by EIR_a_ values of single years from the LMM_2010_ runs.

*Assessment of epidemic malaria risk.* Malaria epidemics are usually defined as an increase in the asexual parasite ratio (i.e., the infection rate) beyond that normally experienced (Macdonald 1957). Epidemics occur in usually malaria-free areas, or they result from significant changes in the normally experienced intraseasonal variation of infection (Kiszewski and Teklehaimanot 2004). In either case, epidemics lead to a marked increase of PR_<15_ and are likely to cause a high year-to-year variability of PR_<15_. Endemic malaria areas with high parasite ratios, however, can also reveal a strong interannual variability of PR_<15_ [denoted as σ(PR_<15_)]. In fact, this year-to-year variability must be understood in the context of its average value. Therefore, we used the coefficient of variation (*c*_v_) of PR_<15_, defined as the ratio of the standard deviation (σ) to the mean (μ) of this quantity, for the risk assessment of malaria epidemics. The usefulness of *c*_v_ is limited when the mean is near zero. Therefore, we did not calculate *c*_v_ values in areas where μ(PR_<15_) reached values of ≤ 0.5%.

## Results

*Present-day malaria distribution (1960–2000).* Spread of malaria transmission. The simulated spread of malaria under present-day climate conditions (1960–2000) is restricted by desert areas and highland regions (Figure 1). We simulated the highest EIR_a_ values and the strongest year-to-year variability for equatorial Africa and the southwest of Cameroon (~ 3°N, 10°E; Figure 1A,B). These are areas with high annual rainfall but not excessive precipitation. For the Congo Basin (centered around 0°N, 22°E), the LMM_2010_ simulates year-round transmission [Figure 1C; for the definition of the malaria season, see Supplemental Material (http://dx.doi.org/10.1289/ehp.1103681); note that months during which monthly EIR is ≥ 0.01 are considered malaria transmission months]. The simulated length of the malaria season (Seas) significantly shortens toward the Sahara and southern Africa, and the highest σ(Seas) values are modeled for East Africa (Figure 1D). The year-to-year variability of PR_<15_ is strongest in low-transmission areas such as the Sahel (Figure 1F).

There is a marked influence of mountainous areas on malaria distribution. In West Africa, projected temperatures ≤ 20°C in the Adamawa Plateau and the Jos Plateau lead to lower transmission (Figure 1A), shorter and delayed malaria seasons [Figure 1C; see also Supplemental Material, Figure 4 (http://dx.doi.org/10.1289/ehp.1103681)], and diminished infection rates (Figure 1E). In East Africa, the presence of highlands causes a complex pattern of malaria distribution. Temperatures in these regions reduce or disrupt transmission, and dry conditions along the Horn of Africa prohibit the simulated spread of malaria (Figure 1; see also Supplemental Material, Figures 1, 2).

We quantitatively compared the simulated, 41-year mean PR_<15_ values with the predicted spatial distribution of *P. falciparum* malaria endemicity of 2007 from the Malaria Atlas Project (MAP; Hay et al. 2009). The geographic malaria extent of the integrated weather–disease model is comparable to that of the MAP model. Most differences regarding the values of parasite ratios vanish when the uncertainty of the MAP model is considered. Differences are found for the northeastern part of Somalia, where the integrated weather–disease model underestimates the malaria occurrence. In addition, the model overestimates PR_<15_ in parts of Senegal, Chad, Sudan, Ethiopia, and Kenya, which is likely attributed to nonmeteorological factors such as malaria control [see Supplemental Material, Figure 3 (http://dx.doi.org/10.1289/ehp.1103681); for details on the validation, see Supplemental Material].

**Figure 3:**
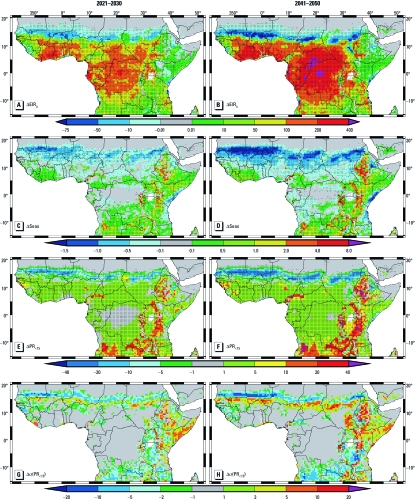
LMM_2010_ and S_2005_ model-projected malaria changes relative to the present-day modeled distribution (1960–2000). (*A*–*D*) illustrate the LMM_2010_-simulated changes in EIR_a_ (*A* and *B*) and Seas (*C* and *D*). (*E*–*H*) S_2005_ model-projected changes in PR_<15_ (*E* and *F*) and σ(PR_<15_) (*G*,*H*). The values are for 2021–2030 (*A*,*C*,*E*,*G*) and 2041–2050 (*B*,*D*,*F*,*H*) compared with the A1B scenario. For *A*–*F*, the values that are statistically significant at the 5% level according to the Wilcoxon–Mann–Whitney rank-sum test (Helsel and Hirsch 2002) are marked by the black dots. The value scales for *A and B *are infectious bites per human per year, *C* and *D *are months, and *E*–*H *are percentages.

Epidemic malaria risk. We detected epidemic malaria risk areas by an artificial threshold of *c*_v_(PR_<15_) ≥ 60%, ensuring that PR_<15_ varies substantially from year to year. Epidemic risk was found along a strip within the Sahelian zone between 13°N and 18°N (Figure 2A), a region well known for unstable malaria transmission (Kiszewski and Teklehaimanot 2004; Snow et al. 1999). In various years between 1960 and 2000, malaria transmission is absent in the LMM_2010_ simulations in the northern part of this zone (Figure 2, black and white dots).

The integrated weather–disease model indicates potential epidemic risk for large parts of the Greater Horn of Africa (Figure 2A). Marginal malaria areas are found either in arid climates or in regions exhibiting temperatures only slightly above the sporogonic temperature threshold of 16–18°C (Detinova 1962). The S_2005_ model correctly identifies the East African highlands as epidemic-prone areas (compare Bonora et al. 2001; Loevinsohn 1994). Highland epidemic risk is concentrated in parts of Ethiopia, western Kenya, southwestern Uganda, Burundi, and the northern part of the Eastern Arc Mountains in Tanzania (compare Cox et al. 1999; see their figure 8.2).

Malaria projections for the 2020s and 2040s: The Sahel. The projected precipitation decline forces a significant decrease of malaria transmission in the Sahel (Figure 3A,B). Transmission rates decrease north of about 13°N, and the seasonality markedly shortens in the scenarios [Figure 3C,D; for an illustration of the length of the main transmission season, the month of the maximum transmission, and the start and end month of the malaria season regarding 1960–2000, 2021–2030, and 2041–2050, see Supplemental Material, Figures 4–6 (http://dx.doi.org/10.1289/ehp.1103681)]. The reduced transmission further translates into a decrease in PR_<15_ (Figure 3E,F). North of about 15°N, malaria infection within humans either fully vanishes or is significantly reduced under the future climate.

Besides the withdrawal of malaria transmission along the Sahara fringe, a change occurs in the year-to-year variability of PR_<15_. Between about 15°N and 18°N the frequency of malaria occurrence is reduced (Figure 2B,C). Compared with 1960–2000, the area of potential epidemic risk is shifted toward the south by about 1–2° (compare Figure 2A). North of about 16°N, malaria epidemics are projected to become less likely. However, the frequency of epidemics is expected to increase farther south in currently more densely populated territories.

Further parts of West Africa. South of the Sahel the decline in precipitation is beneficial for the growth of the mosquito population, which causes higher EIR_a_ values (Figure 3A,B). Under these modified climatic conditions, the LMM_2010_ simulates a reduced flushing of breeding habitats. The start of the malaria season is delayed and ceases earlier under the malaria projections, except for areas between Liberia and Ghana [centered around 7°N, 5°W; Figure 3C,D; Supplemental Material, Figures 4–6 (http://dx.doi.org/10.1289/ehp.1103681)].

We simulated the strongest PR_<15_ increases in West Africa for the Adamawa Plateau (~ 7°N, 12°E) and Jos Plateau (at ~ 10°N, 8°E; Figure 3E,F), resulting from the marked temperature increase.

Greater Horn of Africa. Significantly higher temperatures and slightly higher rainfall [Supplemental Material, Figures 1, 2 (http://dx.doi.org/10.1289/ehp.1103681)] lead to a small or moderate increase in malaria transmission in East Africa (Figure 3A,B). Because of the nonlinear increase of PR_<15_ at low EIR_a_ values (compare Smith et al. 2005, their figure 1), the small rise of EIR_a_ causes a substantial growth of PR_<15_ in formerly epidemic-prone areas. As a result, the spread of malaria is markedly increased in highland territories and in arid and semiarid areas of East Africa (Figure 3A–F).

We simulated the strongest parasite ratio increase for the Ethiopian Highlands, the Eastern Arc Mountains, and parts of the Western Rift Valley. Some grid boxes of the western Kenyan highlands are newly affected, despite the fact that these territories are higher than 2,000 m in the topography of REMO (Figure 3E,F). The changed weather conditions under the future climate projections lead to a notable prolongation of malaria transmission and an earlier start and later end of the season [Figure 3C,D; Supplemental Material, Figures 4–6 (http://dx.doi.org/10.1289/ehp.1103681)].

In East Africa, the interannual variability of the parasite ratio is markedly modified under the future climate (Figure 3G,H). Areas where malaria transmission is projected to become more stable or instable are often located side by side and lead to changes in the epidemic potential (Figure 2).

The change in the epidemic potential in highlands depends on elevation. Analysis of disease transmission against altitude for the Ethiopian Highlands as well as elevated locations in Equatorial East Africa illustrates this fact [Supplemental Material, Figure 7 (http://dx.doi.org/10.1289/ehp.1103681)]. For most grid boxes, the malaria transmission stabilizes in the future projections below about 1,600–2,000 m. In contrast, projections suggest that malaria will climb to formerly malaria-free zones above about 2,000 m, reinforcing the probability of malaria epidemics.

A1B versus B1. Results for scenarios A1B and B1 are similar. However, the changes are generally stronger in scenario A1B than in B1 [for B1, see Supplemental Material, Figures 8–12 (http://dx.doi.org/10.1289/ehp.1103681)]. The uncertainty in the emission scenarios results in the fact that the malaria projections of scenario B1 partly lag those of A1B by one to two decades. For example, in the A1B scenario, epidemic malaria risk above 2,100 m is already high in 2021–2030, when the epidemic risk in B1 reaches levels above 2,000 m [see Supplemental Material, Figures 7, 12 (http://dx.doi.org/10.1289/ehp.1103681)].

Under scenario A1B, malaria transmission disappears in 2041–2050 for an area of about 229,000 km^2^ (74 grid boxes) in the Sahel and appears for the first time in an area of approximately 220,000 km^2^ (71 grid boxes) in the East African highlands compared with 1960–2000 (Figure 2A,C; former areas, dots transfer to crosses; new areas, crosses transfer to dots). Under the B1 scenario, former and new malaria areas for B1 are projected to be about 108,500 km^2^ (35 grid boxes) and 173,500 km^2^ (56 grid boxes), respectively.

## Discussion

The main aim of the present study was to assess the malaria risk in Africa under the present and future climates. For the first time, we carried out malaria projections for Africa that were based on a high-resolution RCM data set taking both greenhouse-gas and LUC changes into account.

*Climate projections.* The advantages of using input data from REMO are as follows:

RCMs have a much higher spatial resolution than do GCMs, which enable RCMs to include local forcing such as from the topography or land surface (Giorgi et al. 2009).
REMO has been shown to reproduce the basic features of African climate in a realistic way, including important rain-bearing atmospheric processes that are usually underrepresented in GCMs (Paeth et al. 2005). REMO simulates such processes as African easterly waves, the African easterly jet, the tropical easterly jet, and the northward shift of the Intertropical Convergence Zone over West Africa.
A scenario of future land-use changes was taken into account. Man-made land-cover changes represent an important source of predictability for the climate in sub-Saharan Africa, especially at time scales of several decades into the future (Paeth et al. 2009).

Much smaller spatial structures can be studied by the 0.5° resolution of REMO than by the use of GCMs. For example, various highland territories can now be studied in more detail. Of course, REMO does not exactly reproduce the relief of mountains with rapid topographic changes [see the discussion in Omumbo et al. (2011) regarding temperature trends in highlands]. Instead of referring to specific areas, we analyzed the general effect of altitude on the epidemic risk for different areas [see Supplemental Material, Figures 7, 12 (http://dx.doi.org/10.1289/ehp.1103681)]. The results of such an analysis also enable decision makers to assess malaria changes for highland territories.

The key feature of the REMO scenarios is the LUC change. Such changes are at present largely not included in state-of-the-art climate models. The inclusion of land-use changes accelerates the precipitation decline by nearly three decades over West Africa (Druyan 2010). This decline results in projected changes in the epidemic potential well before 2050, which may be of larger concern for decision makers than changes projected at the end of the 21st century. Because of these factors, we believe that the more realistic boundary weather conditions from REMO with a high spatial resolution contribute considerably to the predictability of the malaria assessment made in this study.

The atmospheric basis of the malaria projections covers two different climate scenarios from a single RCM. Ideally, assessment of the malaria risk should be based on projections from different climate models. The use of multimodel RCM data from the ENSEMBLES (Ensemble-Based Predictions of Climate Changes and Their Impacts) and the AMMA (African Monsoon Multidisciplinary Analysis) projects, for example, could improve the assessment of the malaria future (Paeth et al. 2011).

*Malaria projections.* The spread of malaria is realistically reproduced by the integrated weather–disease model for Africa. Moreover, the simulated parasite ratio is comparable to MAP data for large parts of tropical Africa, when the uncertainty of the MAP model is considered. However, we detected substantial overestimations of parasite ratios for various areas where malaria control might have reduced infection. Note that the integrated model was driven by daily meteorology variables from REMO, and the parameter setting and model structure (see Ermert et al. 2011a, 2011b) do not include other factors such as malaria control measures.

Various other published malaria distribution maps also correspond to the simulated malaria spread (e.g., Craig et al. 1999; Kleinschmidt et al. 2001; Rogers et al. 2002). Contrary to our simulations, the transmission intensities and infection rates provided by Gemperli et al. (2006) and Kleinschmidt et al. (2001), respectively, reveal partly spotted malaria transmission and infection maps. In fact, their results suffer from the neglected interannual variability of malaria.

We confirmed the general expectations of the impact of altered temperature and precipitation patterns under the future climate on the spread of malaria. The IPCC already concluded that climate change will be associated both with geographical expansions and with contractions of the malaria distribution (Confalonieri et al. 2007). Areas most probably affected are parts of the Sahel, the Horn of Africa, or various highland territories. We projected that malaria areas will disappear in the northern Sahel and that the disease distribution will expand in highlands. Our study projects seasonality changes for various endemic malaria areas.

Rainfall projections for the Sahel are at present uncertain (Druyan 2010; Paeth et al. 2011). This leads to an uncertainty of the projected transmission changes in the Sahel. Nevertheless, Peterson (2009) and Thomas et al. (2004) also projected a reduction in malaria transmission, although due to warmer temperatures. Therefore, the reduction of transmission is one plausible picture of the future spread of malaria in the Sahel. We project more densely populated zones in the southern parts of the Sahel to turn into epidemic-prone areas.

In contrast, it is generally accepted that climate change will increase the spread of malaria in altitude in highland areas. Ebi et al. (2005), for example, projected an increase in the malaria suitability of Zimbabwe highlands. Also, Peterson (2009) and Rogers and Randolph (2000) predicted an increase in malaria suitability of East African highlands by 2050. One key result of our study is the different response of epidemic risk to different altitudes in highland areas. As indicated by Githeko et al. (2000), malaria is projected to become stable below about 1,600–2,000 m. In contrast, formerly unsuitable territories above 2,000 m are projected to turn into epidemic-prone areas.

The present study aimed only at assessing potential weather-driven changes accounting for LUC changes in the environmental suitability of malaria. For example, human interventions such as vector control measures or the use of insecticide treated bed nets were not considered. The model neglects the relative abundance of different malaria vectors (Lindsay et al. 1998) and variable human population densities such as rural versus urban sites (e.g., Keiser et al. 2004). We did not take into account indirect effects of climate change on the malaria spread (Saugeon et al. 2009) such as migration of people. It must be noted, therefore, that the malaria projections will not necessarily translate into realized changes in malaria risk.

## Conclusions

The present study might enter new weather–malaria modeling territory for tropical Africa. For the first time, malaria projections are based on a 0.5° resolution and include greenhouse-gas and LUC changes. The risk assessment focuses solely on daily temperatures and precipitation data and hence assumes no future human-imposed constraints on the disease. According to the projections of the integrated weather–disease model, the distribution of malaria epidemics will be strongly altered in the years approaching 2050. The inclusion of land-use changes causes an accelerated prominent precipitation decline, resulting in earlier malaria changes, which needs to be considered by malaria control programs. Epidemic-malaria risk will be shifted southward in the Sahel and toward higher altitudes in highland territories.

## Supplemental Material

(12.5 MB) PDF
