# Supplementary material for: The Impact of Regional Climate Change on Malaria Risk due to Greenhouse Forcing and Land-Use Changes in Tropical Africa
Source: Environ Health Perspect. 2011 Sep 7;120(1):77–84. doi: 10.1289/ehp.1103681 (PMC3261943; doi:10.1289/ehp.1103681)
Supplement: (12.5 MB) PDF [file ehp.1103681.s001.pdf]

Supplemental Material

The Impact of Regional Climate Change due to Greenhouse Forcing and Land-Use Changes on Malaria Risk in Tropical Africa

Volker Ermert[1], Andreas H. Fink[1], Andrew P. Morse[2], and Heiko Paeth[3]

[1] Institute of Geophysics and Meteorology, University of Cologne, Germany
[2] School of Environmental Sciences, University of Liverpool, United Kingdom
[3] Institute of Geography, University of Würzburg, Germany

Table of Contents

1 Definition of the malaria seasonality 2

1.1 Malaria seasonality with regard to single years 2
1.2 Climatological malaria seasonality 3
Supplemental Material, Table 1. Example regarding the definition of the malaria seasonality. 3

2 Validation of the integrated weather-disease model 5

3 Supplemental figures 8

Supplemental Material, Figure 1. REMO projected temperatures. 8
Supplemental Material, Figure 2. REMO projected precipitation. 9
Supplemental Material, Figure 3. Validation of the integrated weather-disease model. 10
Supplemental Material, Figure 4. Malaria seasonality of the LMM_{2010} (1960-2000). 11
Supplemental Material, Figure 5. A1B: LMM_{2010} projected malaria seasonality (2021-2030). 12
Supplemental Material, Figure 6. A1B: LMM_{2010} projected malaria seasonality (2041-2050). 13
Supplemental Material, Figure 7. A1B: Effect of altitude on malaria epidemics. 14
Supplemental Material, Figure 8. B1: Risk assessment of malaria epidemics. 15
Supplemental Material, Figure 9. B1: LMM_{2010} and S_{2005} model projected malaria changes. 16
Supplemental Material, Figure 10. B1: LMM_{2010} projected malaria seasonality (2021-2030). 17
Supplemental Material, Figure 11. B1: LMM_{2010} projected malaria seasonality (2041-2050). 18
Supplemental Material, Figure 12. B1: Effect of altitude on malaria epidemics. 19

References 20

1 Definition of the malaria seasonality

1.1 Malaria seasonality with regard to single years

The definition of the malaria season was already described by Ermert et al. 2011b (see also Ermert 2010) and 'is based on the *monthly entomological inoculation rate* (EIR_m'; i.e. the number of infectious bits per human per month), 'which is observed in field studies. The introduction of the malaria parasite into the' *Liverpool Malaria Model version of 2010* (LMM_{2010}; Ermert et al. 2011a,b) 'is assured by a constant influx of new infectious mosquitoes resulting in transmission values that might exceed those of low transmission areas. In order to compare *truly* modelled EIR_m values with field observations, the artificial EIR_m values were removed via two separate LMM_{2010} runs. The standard run results in a mixture of bites from the added infectious mosquitoes and those which were infected when they bit infectious humans in the simulation. The artificial EIR_m values were produced in a second run when only the added infectious mosquitoes were biting in the model, achieved by setting the *number of produced eggs per female mosquito* ($\#E_p$) to zero. Here, the oviposition of *Anopheles* females is prohibited and the mosquito population is therefore not able to grow. The subtraction of the EIR_m values of the second run from that of the standard run results finally in the elimination of artificial EIR_m values' (Ermert et al. 2011b).

'In field studies, the malaria season is mostly determined on a monthly scale and is usually referred to months with EIR_m values above zero (Fontenille et al. 1997). For this reason, the *start of the malaria season* ($SSeas$) is defined by the first month with an EIR_m value of at least 0.01 infectious bites per human. According to the formulation of the model, the arbitrary EIR_m value of 0.01 means that during a month at least one out of the 100 modelled humans is bitten by an infectious mosquito. In West Africa, the malaria season usually ends when the number of mosquitoes decreases at the end of the rainy season. Therefore, mosquito biting is reduced to such low numbers that the EIR_m values are reduced to zero indicating that transmission has ceased. Consequently, the last month during the transmission period defintes the *end of the malaria season* ($ESeas$). Some individual years also reveal year-round or even no malaria transmission for certain locations. The *length of the malaria season* ($Seas$) is therefore the number of months with EIR_m reaching at least 0.01 infectious bites. For each site or grid box additionally the *length of the main transmission season* ($MSeas$) is defined as the number of months in which 75% of the *annual entomological inoculation rate* (EIR_a'; i.e. the number of infectious bites per human per year) 'is transmitted, an index which was used by Hay et al. (2000). Where possible the *month with the maximum malaria transmission* ($XSeas$) is identified as the month with the highest EIR_m value' (Ermert et al. 2011b).

'The use of the transmission threshold of 0.01 infectious mosquito bites per human per month ensures the attainment of a reasonable transmission level in the model that can be compared with observations from the literature. However, the definition of the malaria season in the model might not be directly comparable to field studies since observations are subject to a certain detection limit. That is due to the fact that field experiments do not continuously measure biting rates (at best two times in each week of the field

campaign) and that these field studies do not account for every human of the population of the study site' (Ermert et al. 2011b).

1.2 Climatological malaria seasonality

The definition of a climatological start and end of the malaria season is more complex, especially in case of two distinct malaria seasons. Regarding a long time period such as 100 years, the start and end of malaria transmission usually takes place within certain months of a year. For example, transmission in the Sahel typically starts between June and August (e.g. Fontenille et al. 1997). However, variable atmospheric conditions such as an early start of the rainy season might cause unusual transmission seasons. Such events complicate the determination of typical characteristics of the malaria season in particular in areas with two distinct malaria seasons. The artificial example (Supplemental Material, Table 1), for instance, shows 153 starts of the malaria season between May and January. In this example, obviously two malaria seasons are present. The first season begins between July and August and the second season starts most frequently in December, but also starts in January. Due to the infrequent, but non-zero occurrence of malaria season starts between September and November, an objective and direct determination of the two typical season starts is impossible. In areas with two malaria seasons the definition of the two distinct seasons might, therefore, not be straightforward. For this reason, only typical start and end months of particular malaria seasons are considered by the subsequent method. The definition of the start of the malaria season is explained in the following. The determination of $ESeas$ as well as $XSeas$ is fully analogous to the definition of $SSeas$:

**Analysis**	**J**	**F**	**M**	**A**	**M**	**J**	**J**	**A**	**S**	**O**	**N**	**D**
Number of events: $fr(m)$	24	0	0	0	3	10	26	14	1	1	2	72
Criteria A: $fr(m-1)+fr(m)$	96	24	0	0	0	13	36	40	15	2	3	74
Criteria B: $fr(m)+fr(m+1)$	24	0	0	0	13	36	40	15	2	3	74	96
Threshold	25	25	25	25	25	25	25	25	25	25	25	25
Fulfilled criteria (A or B)	1	0	0	0	0	1	1	1	0	0	1	1

Supplemental Material, Table 1: Artificial example relative to the determination of the season start for a period of 100 years. Within the time frame 153 season starts are identifiable. Criteria A and B require at least 26 events for the considered month and the preceding as well as subsequent month, respectively. The periods June-August as well as November-January are selected by the procedure (indicated by '1'; see last row). The average of the first season is hence month 7.08 ($= (10 \cdot 6 + 26 \cdot 7 + 14 \cdot 8)/50$; i.e. about July) and that for the second season is month 12.22 ($= (2 \cdot 11 + 72 \cdot 12 + 24 \cdot 13)/98$); i.e. about December).

In a first step, the *frequency* (fr) of the season start within a certain month is computed. A given month (m) is only considered when a frequent start of the malaria season is found in the preceding month and the month itself or in the given month and the subsequent month. The frequency of such events must exceed 25% (criterion A: $fr(m-1) + fr(m) > 0.25 \cdot n_y$; criterion B: $fr(m) + fr(m+1) > 0.25 \cdot n_y$; n_y: number

of years of the period). For the calculation of the mean or standard deviation of the season start only those months are retained that fulfil either criterion A or B (see also the example in Supplemental Material, Table 1). Note that several start periods during the year might have to be considered. These time periods are simply separated by months that do not accomplish criteria A and B.

Instead of using months, the categories 'U', 'V', and 'C' are used in the following cases: Category 'U' is applied when transmission occurs infrequently ($\sum fr(m) \leq 0.25 \cdot n_y$). Category 'V' is used when $SSeas$, $ESeas$, or $XSeas$ are simulated more often ($\sum fr(m) > 0.25 \cdot n_y$) but are not sufficiently clustered (criteria A and B are not fulfilled). Such a situation might be characterised as a variable malaria season. Category 'C' is utilized in terms of $SSeas$ and $ESeas$ when the malaria transmission is frequently year-round in the simulation (criteria A and B are not fulfilled and $Seas > 11$ months).

Due to the chosen procedure it is possible that a different number of periods are found for $SSeas$, $ESeas$, and $XSeas$. For example, for some grid boxes in East Africa two malaria seasons are merged by the used method or only one season start (end) is found despite most frequently simulated two season ends (starts).

2 Validation of the integrated weather-disease model

As already noted, the LMM_{2010} has been validated by the usage of data from 34 West African weather stations and malaria observations from field sites in their vicinity. The main result of this validation was that the LMM_{2010} is able to reproduce *annual Entomological Inoculation Rates* (EIR_a; i.e. the number of infectious bites per human per month) in the range of field observations. In addition, the *Plasmodium falciparum infection model of Smith et al. (2005)* (termed S_{2005} model) was built by using paired EIR_a and *asexual parasite ratios of children under 15 years of age* ($PR_{<15}$) values, which should ensure the calculation of realistic infection rates of children. It is true that the simulated two-dimensional malaria distribution lacks a quantitative verification, which is now provided.

The integrated weather-disease model is compared with an existing modelled malaria map. This provides a comparison between the simulated $PR_{<15}$ values from the S_{2005} model and that of the predicted *Plasmodium falciparum* parasite ratio of children between 2 and 10 years ($PfPR_{2-10}$) from the *Malaria Atlas Project* (MAP; Hay et al. 2009). Note that this comparison is somewhat problematic since: (i) The map of malaria endemicity of MAP was produced for 2007 and the integrated weather-disease model represents average values with regard to 1960-2000. Due to the fact that there is a strong interannual variability, parasite ratios of single years should normally not been compared to climatological values (here: 1960-2000). Hay et al. (2009) found, for example, that the observations from 2007 were substantially lower than the data from the other considered years (1985-2006). In Senegal, the malaria endemicity was, for instance, much higher in the 1960s than after the following drought conditions, when a substantial malaria decline was observed (e.g. Faye et al. 1995). (ii) Hay et al. (2009) computed the parasite ratio of children between 2 and 10 years, whereas the S_{2005} model is constructed for children under the age of 15 years. Due to the increase of immune individuals with age and the simultaneous decline in the infection rate (e.g. Bekessy et al. 1976), the values from the S_{2005} model should be somewhat lower than that of the MAP model. Despite these two issues the comparison of the two maps enables a further insight in terms of the skill of our integrated weather-disease model.

In order to compare the two maps, the MAP data was aggregated from the 5 km x 5 km latitude-longitude grid to the resolution of the integrated weather-disease model of 0.5°. Differences between the two maps cannot be avoided at the fringe of the malaria area. For example, the malaria gaps (according to an asexual parasite ratio threshold of 0.1%) in the north-east of Somalia included in the MAP data are not captured by the aggregated data (compare Supplemental Material, Figure 3b & c). In general, small gaps such as in the East African highlands are reduced by the 0.5° grid.

In a first step, the geographic distribution is compared between the two malaria distributions. According to the difference plot of the two maps (see Supplemental Material, Figure 3d), two general statements can be made: (i) Both models show about the same geographic distribution of asexual parasite ratios. (ii) The integrated weather-disease model underestimates the territory of malaria endemicity in the Horn of Africa, especially for the north-eastern part of Somalia (see the crosses in Supplemental Material,

Figure 3d). However, also the MAP model partly predicts gaps for North-Eastern Somalia (Supplemental Material, Figure 3b), which disappear in the 0.5° latitude-longitude resolution (Supplemental Material, Figure 3c). Beyond these two major aspects, small differences are found at the fringes of the endemicity area. The distribution of the integrated weather-disease model extends one to two degrees further to the north in various parts of the Sahel (see the dots in Supplemental Material, Figure 3d). This might be realistic since Hay et al. (2009) found higher parasite ratios for 1985-2006 than for 2007. It should be further noted, that malaria will probably be distributed further north under wet atmospheric conditions such as that before 1970. There seem to be also differences between the two maps for the East African highlands and the Adamawa plateau (see the crosses in Supplemental Material, Figure 3d). However, this again is an issue of the different grid resolution. The integrated weather-disease model, for instance, simulates a gap in the parasite ratios for the Adamawa plateau (Western Cameroon), which is evident in the full resolution, but vanishes in the 0.5° aggregated resolution (Supplemental Material, Figure 3a-c).

In a second step, the parasite ratios of the two maps are quantitatively compared using a difference plot. This comparison leads to further three statements: (i) The differences in the values of the parasite ratio disappear in most areas when the uncertainty of the MAP model is considered (Supplemental Material, Figure 3f). However, the integrated weather-disease model produces for most parts of tropical Africa higher parasite ratios than the MAP model. (ii) The simulated parasite ratios of parts of Senegal, Chad, Sudan, Ethiopia, and Kenya from the integrated weather-disease model are too high. For these regions, the parasite ratios are 40% higher than predicted for 2007 by the MAP model and this difference is still present when the uncertainty of the MAP model is taken into account (Supplemental Material, Figure 3f). The integrated weather-disease model either fails to simulate lower parasite ratios in these areas or the values of 2007 are not representative for other years. It is most probable, that other factors than weather and climate conditions such as malaria control measures caused these comparable low $PfPR_{2-10}$ values for 2007. Hay et al. (2009) noted that the malaria endemicity was in general stronger in former years (see also Hay et al. 2009, their Figure One). Note the present study does not account malaria control measures. (iii) The integrated weather-disease model simulates too low parasite ratios in low endemicity areas in comparison to the MAP model (Supplemental Material, Figure 3d). For example, in the northern Sahel the parasite ratios of the integrated weather-disease model are up to 20% lower than that of the MAP model (Supplemental Material, Figure 3d). It must be noted here that the MAP model overestimated the $PfPR_{2-10}$ values of low endemicity areas (Hay et al. 2009) and the difference totally disappears, when the uncertainty of the MAP model is taken into account (Supplemental Material, Figure 3f).

Given the uncertainty in the MAP model and the constraints when comparing parasite ratios from a single year (here: 2007) with average values of a period (here: 1960-2000), it is valid to state that: The geographic malaria extent of the integrated weather-disease model is comparable to that of the MAP model. Most differences in terms of values of the parasite ratio vanish when the uncertainty of the MAP model is considered. Differences are found for the north-eastern part of Somalia, where the model underestimates

the malaria occurrence. The integrated weather-disease model overestimates the malaria prevalence in parts of Senegal, Chad, Sudan, Ethiopia, and Kenya, which is likely attributed to factors such as malaria control.

3 Supplemental figures

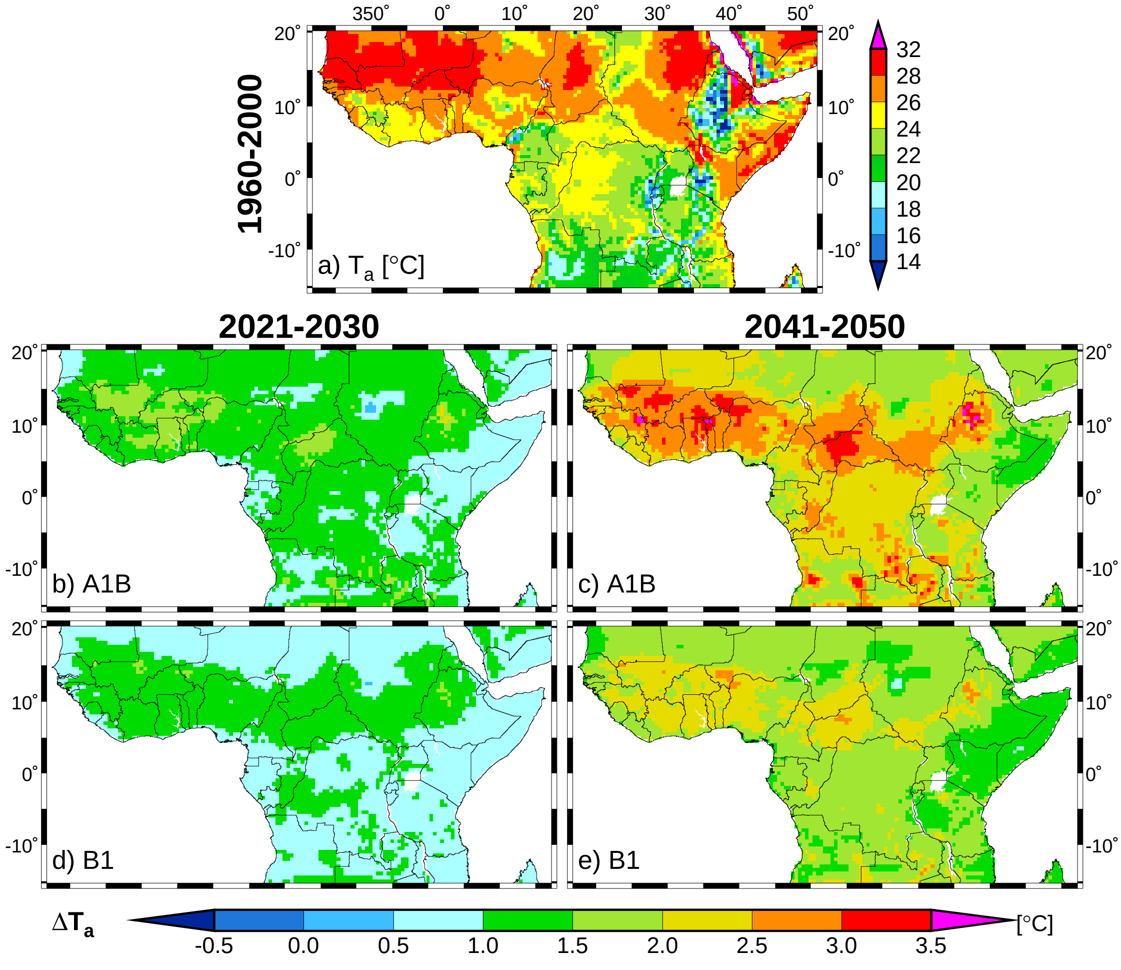

Supplemental Material, Figure 1: Illustration of the bias-corrected *annual mean temperature* (T_a in °C) of REMO for (a) 1960-2000 and for (b-e) differences in T_a (ΔT_a) relative to the present-day climate (1960-2000) for 2021-2030 (b & d) and 2041-2050 (c & e) of the A1B (b & c) and B1 (d & e) scenarios. Note that all displayed grid boxes show statistical significant temperature changes at the 5% level relative to the *Wilcoxon-Mann-Whitney* (WMW) rank-sum test (see e.g. Helsel and Hirsch 2002).

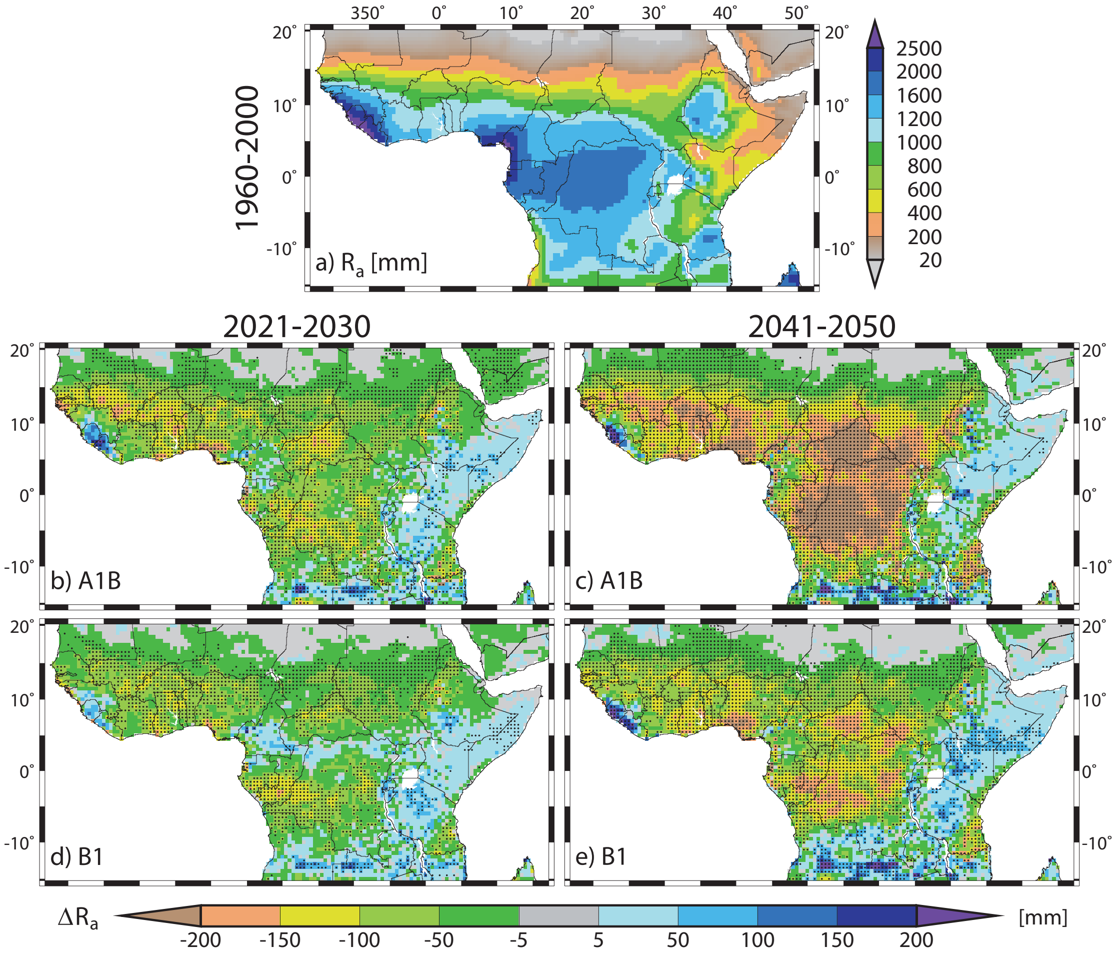

Supplemental Material, Figure 2: Same as Supplemental Material, Figure 1 but for the bias-corrected *annual precipitation amount* (R_a in mm). Values statistical significant at the 5% level according to the WMW rank-sum test are marked by black dots.

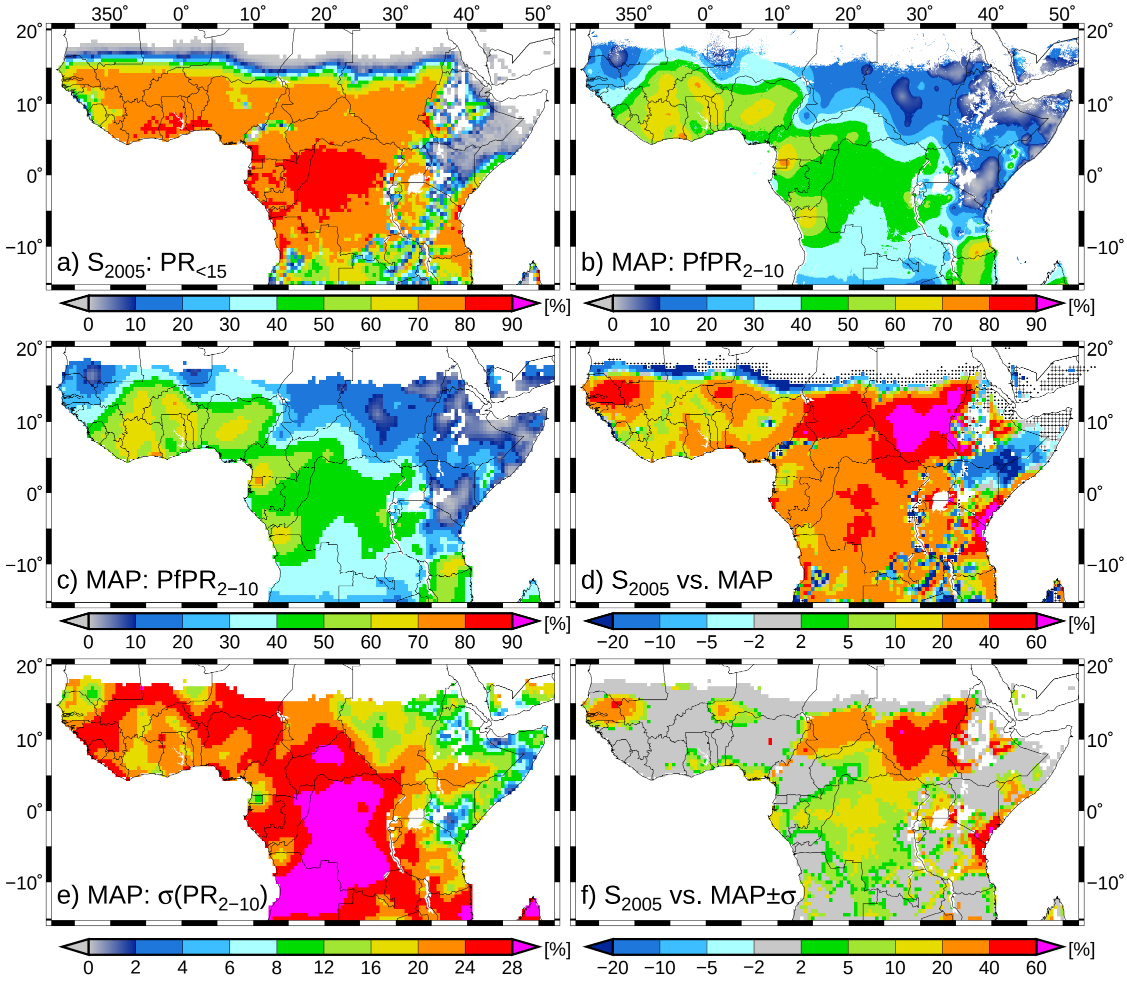

Supplemental Material, Figure 3: Validation of the integrated weather-disease model by the usage of the malaria endemicity map of the *Malaria Atlas Project* (MAP; Hay et al. 2009): (a) S_{2005} model simulated *asexual parasite ratio of children under 15 years of age* ($PR_{<15}$) of 1960-2000. (b) MAP model predicted *Plasmodium falciparum parasite ratio of children between 2 and 10 years* ($PfPR_{2-10}$) of 2007 on the original 5 km x 5 km latitude-longitude resolution. (c) Same as (b) but for a resolution of 0.5°. (d) Difference between the $PR_{<15}$ values of 1960-2000 from the S_{2005} model and the $PfPR_{2-10}$ values of 2007 from the MAP model. Dots indicate those areas where the S_{2005} model simulates $PR_{<15}$ values, which are higher than or equal to 0.1% and where the MAP model predicts $PfPR_{2-10}$ values lower than 0.1%. Crosses mark grid boxes where the MAP model predicts $PfPR_{2-10}$ values, which are higher than or equal to 0.1% and where the S_{2005} model simulates $PR_{<15}$ values lower than 0.1%. (e) Uncertainty (*standard deviation*) of the $PfPR_{2-10}$ values ($\sigma(PR_{2-10})$) of 2007 from the MAP model. (f) Same as (d) but including the uncertainty of the $PfPR_{2-10}$ values of 2007. In all maps those areas are excluded which show $PR_{<15}$ or $PfPR_{2-10}$ values below 0.1%.

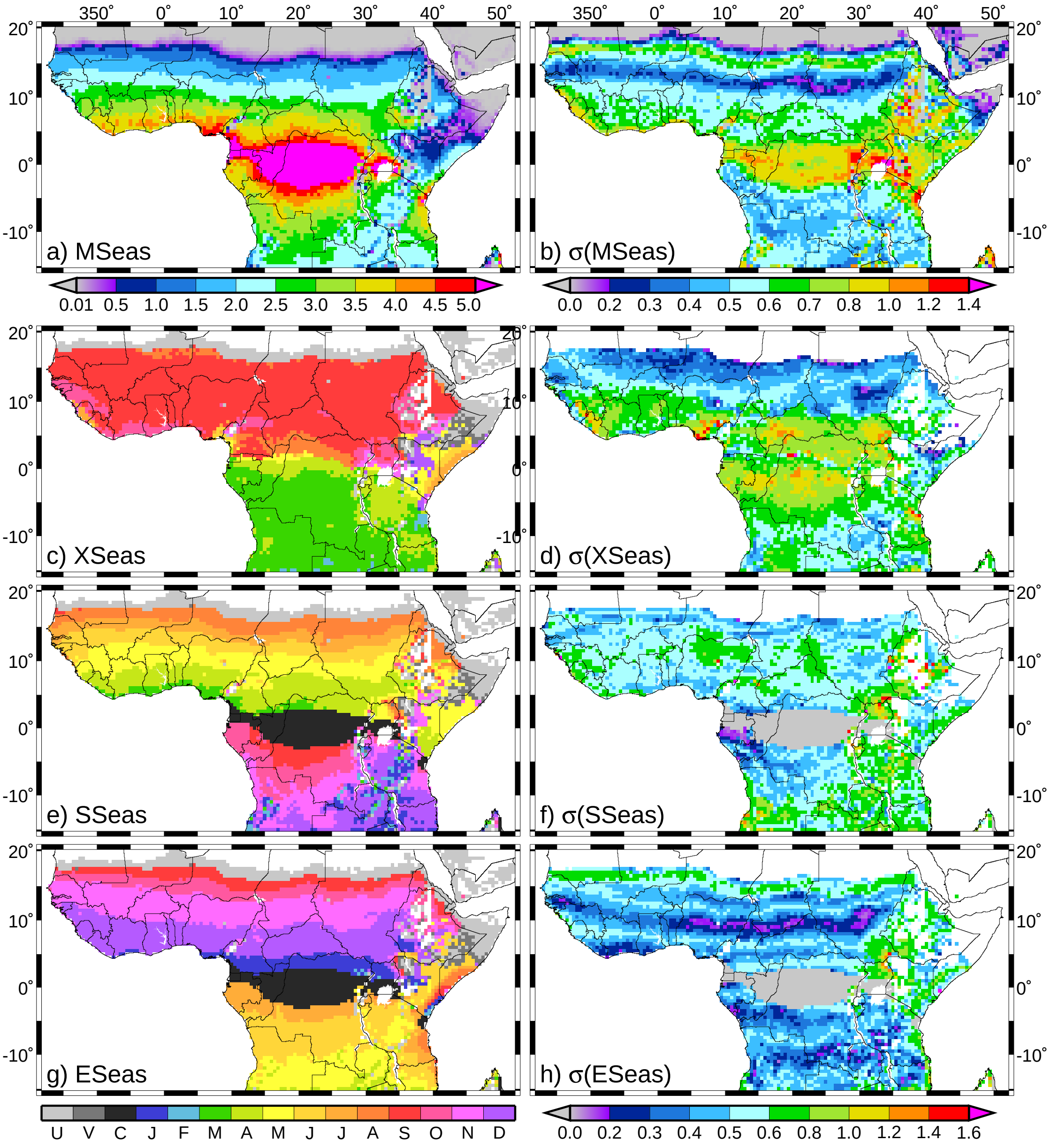

Supplemental Material, Figure 4: LMM_{2010} simulated malaria seasonality (in months) for 1960-2000 based on bias-corrected daily temperatures and precipitation from REMO. Depicted are (a) the *length of the main malaria season* ($MSeas$; in months), (b) the standard deviation of $MSeas$ ($\sigma(MSeas)$), (c) the *month of maximum transmission* ($XSeas$), (d) the standard deviation of $XSeas$ ($\sigma(MSeas)$; in months), (e) the *start month of the malaria season* ($SSeas$), (f) the standard deviation of $SSeas$ ($\sigma(MSeas)$, in months), (g) the *end month of the malaria season* ($ESeas$), and (h) the standard deviation of $ESeas$ ($\sigma(ESeas)$, in months). 'U' indicates areas of unfrequent malaria transmission; 'V' denotes grid boxes with either a rare malaria occurrence or a variable seasonality; 'C' identifies areas of year-round malaria transmission.

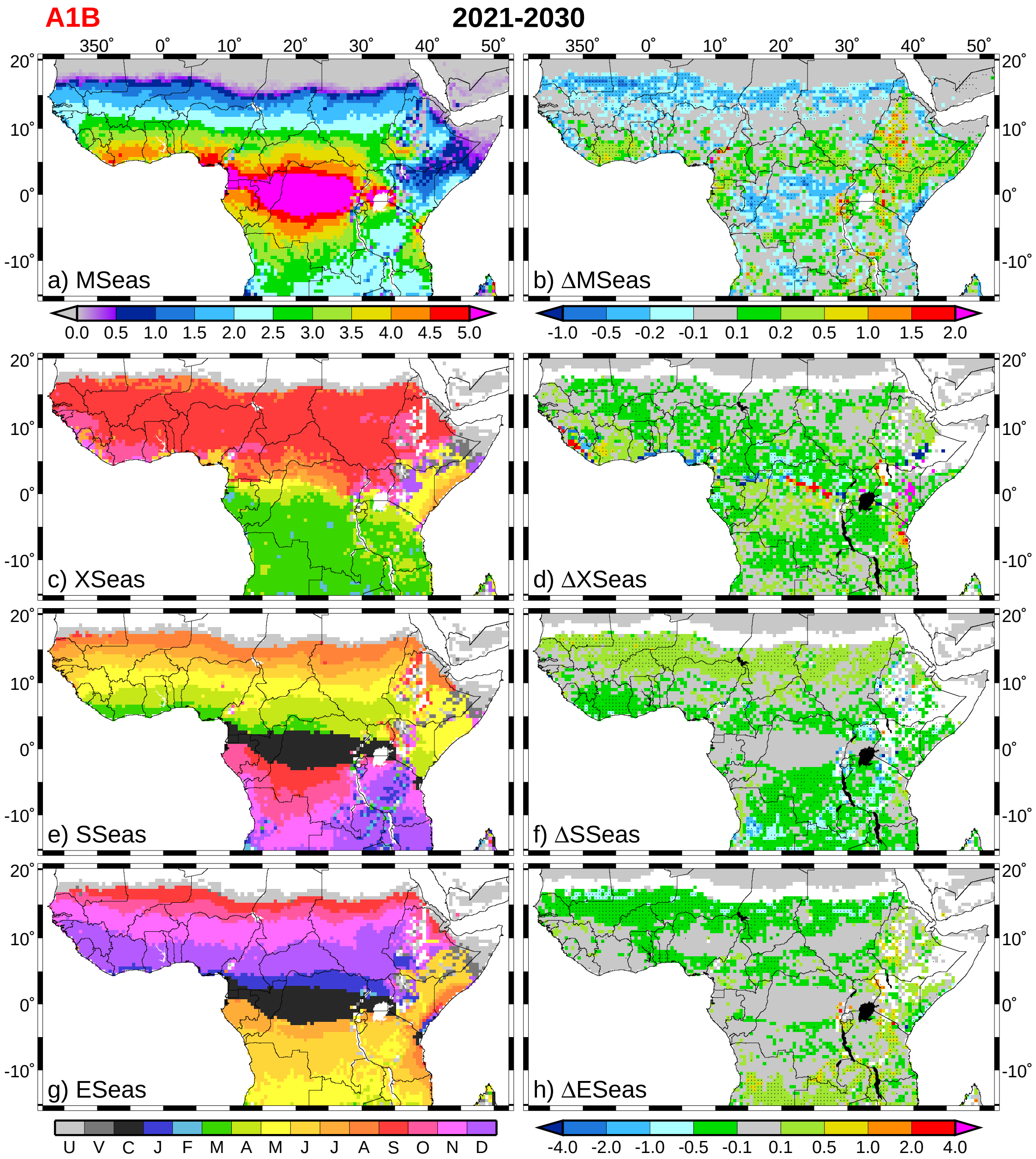

Supplemental Material, Figure 5: LMM_{2010} projected malaria seasonality (in months) for 2021-2030 based on the A1B scenario. Illustrated are absolute values as well as changes (Δ) relative to the present-day climate (1960-2000). Depicted are (a) the *length of the main malaria season* ($MSeas$, in months), (b) $\Delta(MSeas)$, (c) the *month of maximum transmission* ($XSeas$), (d) $\Delta(XSeas)$ (in months), (e) the *start month of the malaria season* ($SSeas$), (f) $\Delta(SSeas)$ (in months), (g) the *end month of the malaria season* ($ESeas$), and (h) $\Delta(ESeas)$ (in months). Note that in case of $XSeas$, $SSeas$, $ESeas$ positive (negative) values stand for a later (earlier) occurrence. 'U' indicates areas of unfrequent malaria transmission, 'V' denotes grid boxes with either a rare malaria occurrence or a variable seasonality, and 'C' identifies areas of year-round malaria transmission. White areas in (d), (f), and (h) refer to areas assigned either for the present-day or future climate to 'U' or 'V'. In the right column, values statistically significant at the 5% level are marked by dots.

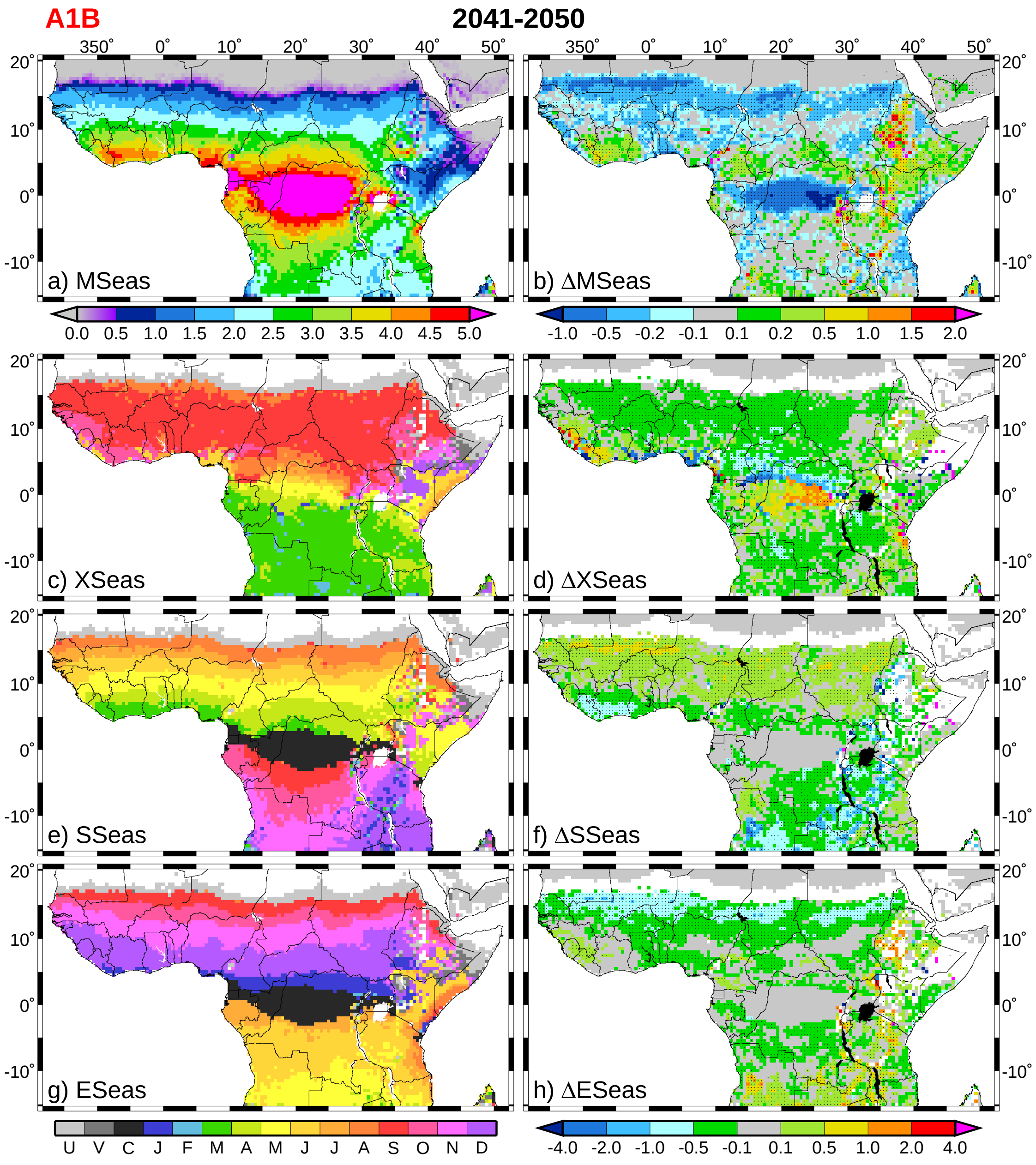

Supplemental Material, Figure 6: Same as Supplemental Material, Figure 5 but for the period 2041-2050.

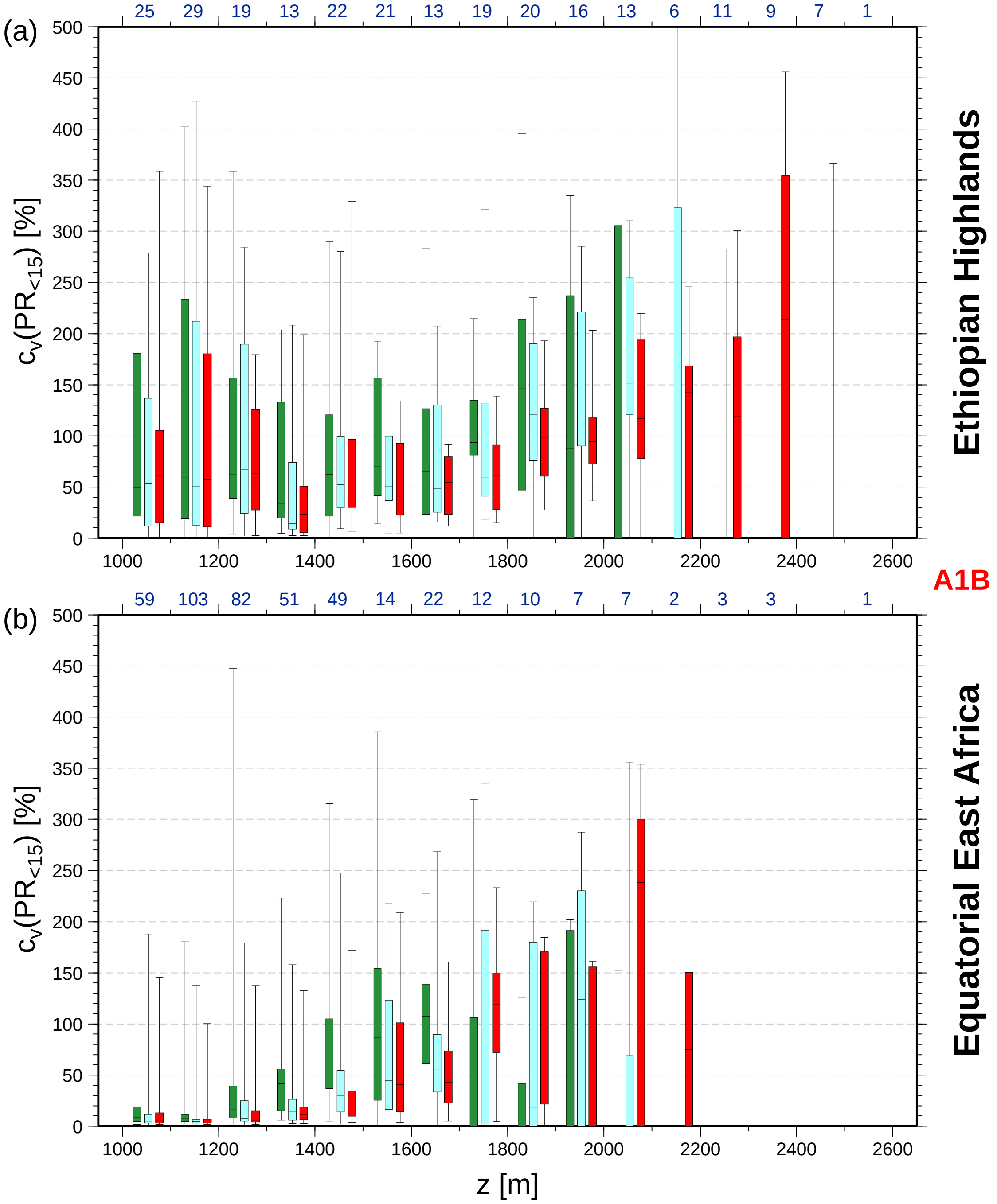

Supplemental Material, Figure 7: Effect of altitude (see abscissa) on the S_{2005} model simulated values of the *coefficient of variation of the asexual parasite ratio of children under 15 years of age* ($c_v(PR_{<15})$; see ordinate) in (a) the Ethiopian Highlands (cf. the framed area north of 4°N in Supplemental Material, Figure 8a) and (b) for Equatorial East Africa (10°S-4°N, 27-38°E) relative to the past period and the A1B scenario. The data from various grid points between height levels of 1000 and 2600 m are grouped within 100 m altitude segments. The box-and-whisker plots represent ensemble average values of the included grid points relative to 1960-2000 (green box plots), 2021-2030 (light blue box plots), and 2041-2050 (red box plots). Blue numbers above (a) and (b) indicate the number of grid points from which the statistic is computed.

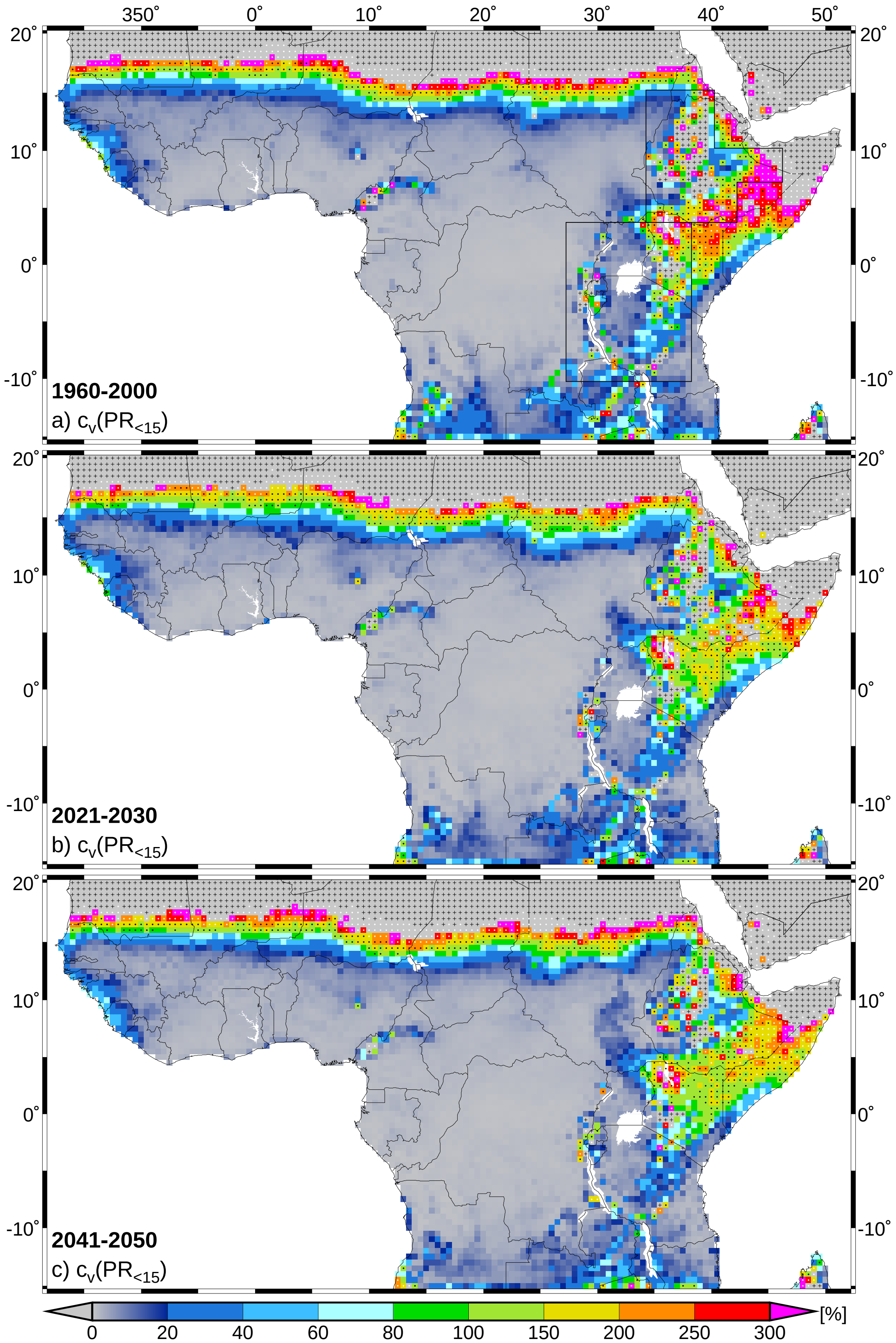

Supplemental Material, Figure 8: Same as Figure 1 but for the B1 scenario.

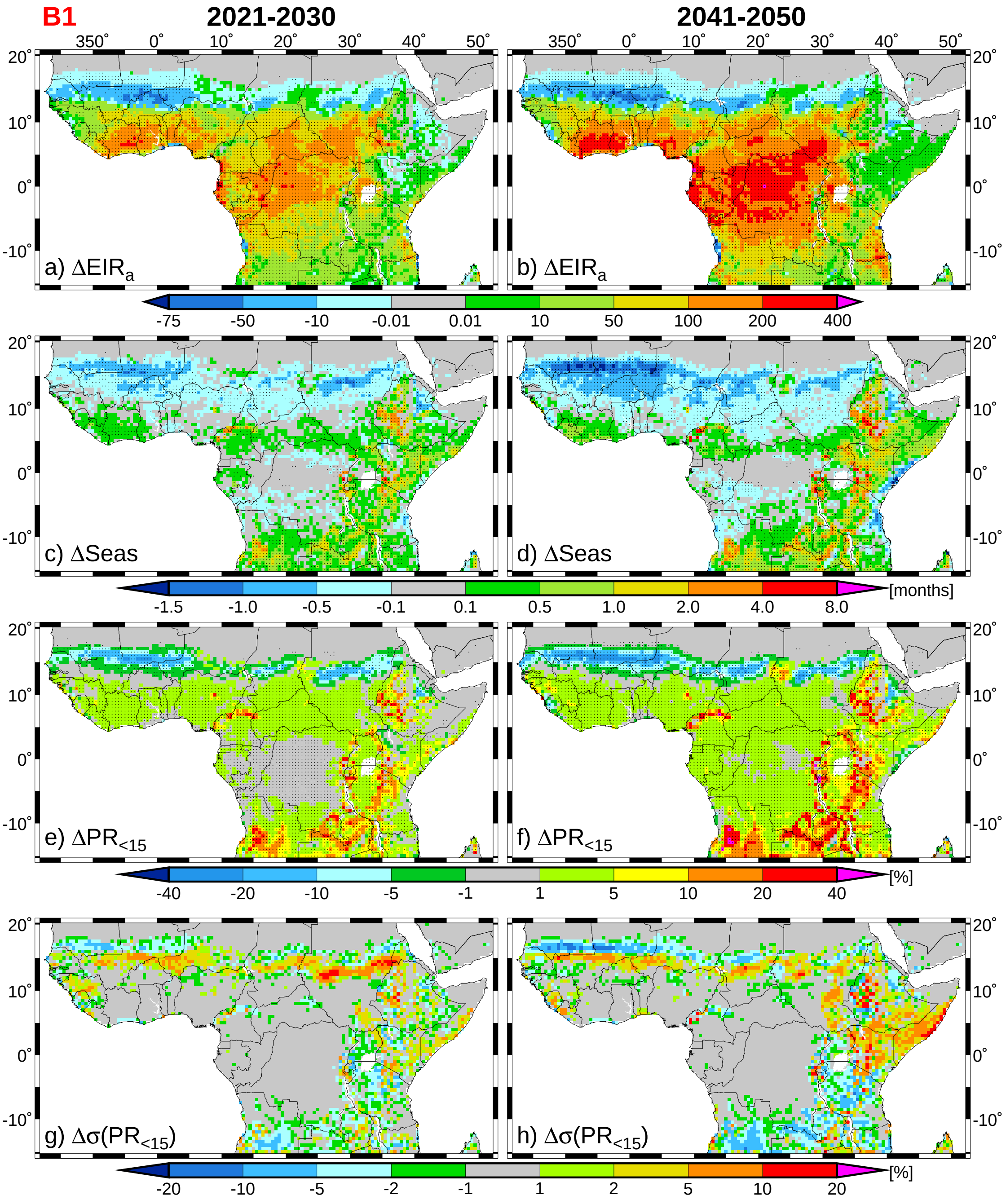

Supplemental Material, Figure 9: Same as Figure 3 but for the B1 scenario.

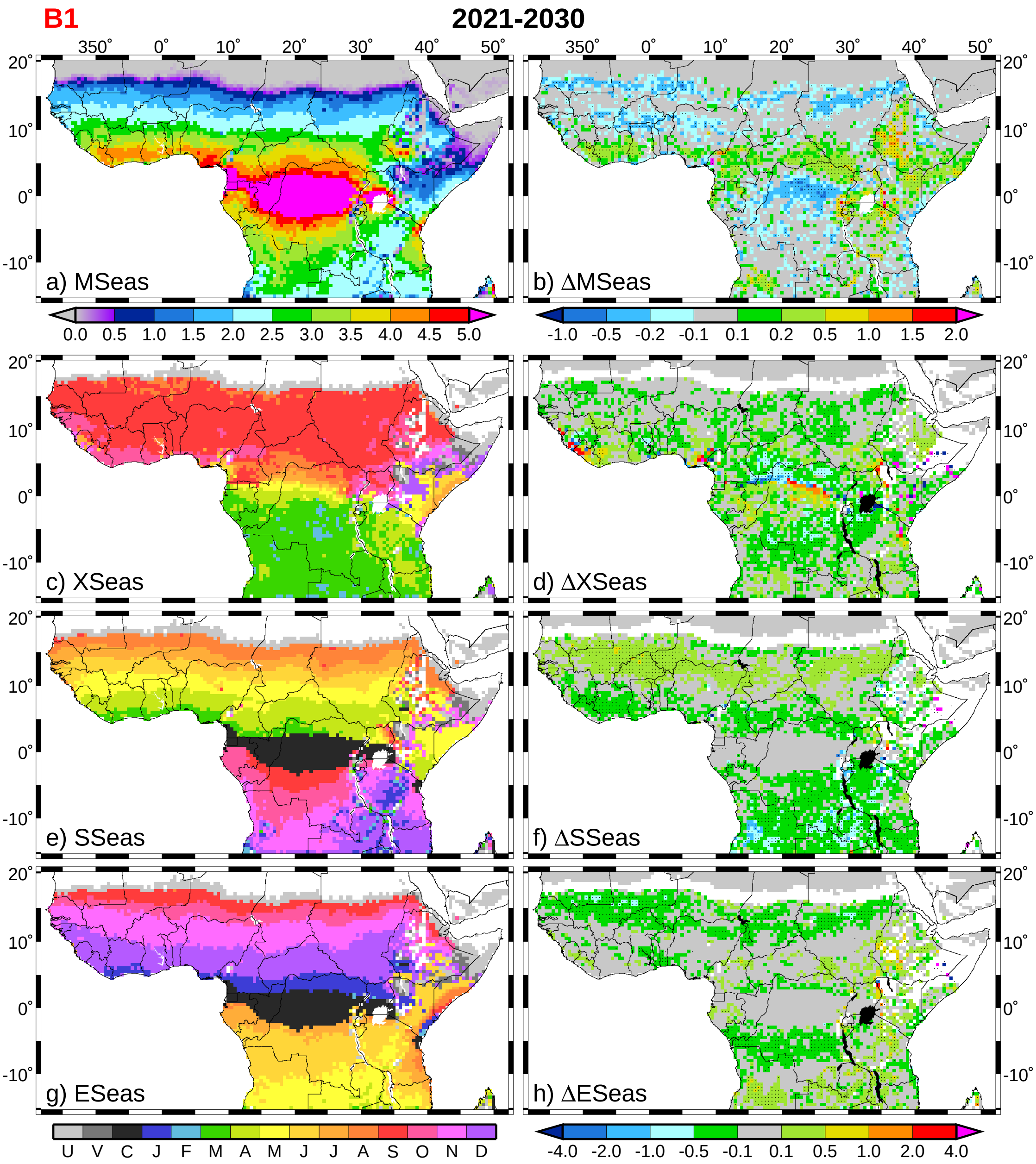

Supplemental Material, Figure 10: Same as Supplemental Material, Figure 5 but for the B1 scenario.

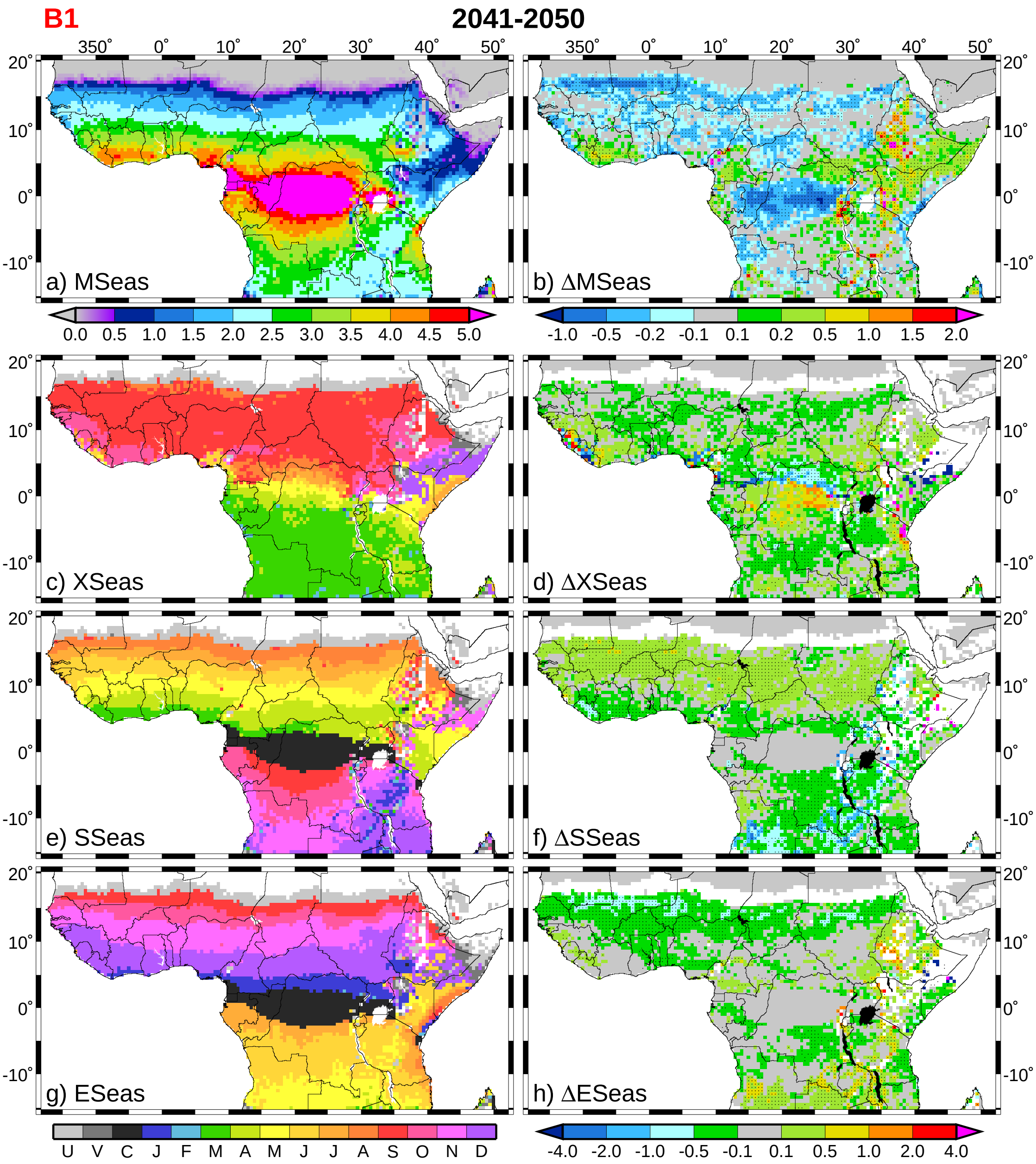

Supplemental Material, Figure 11: Same as Supplemental Material, Figure 6 but for the B1 scenario.

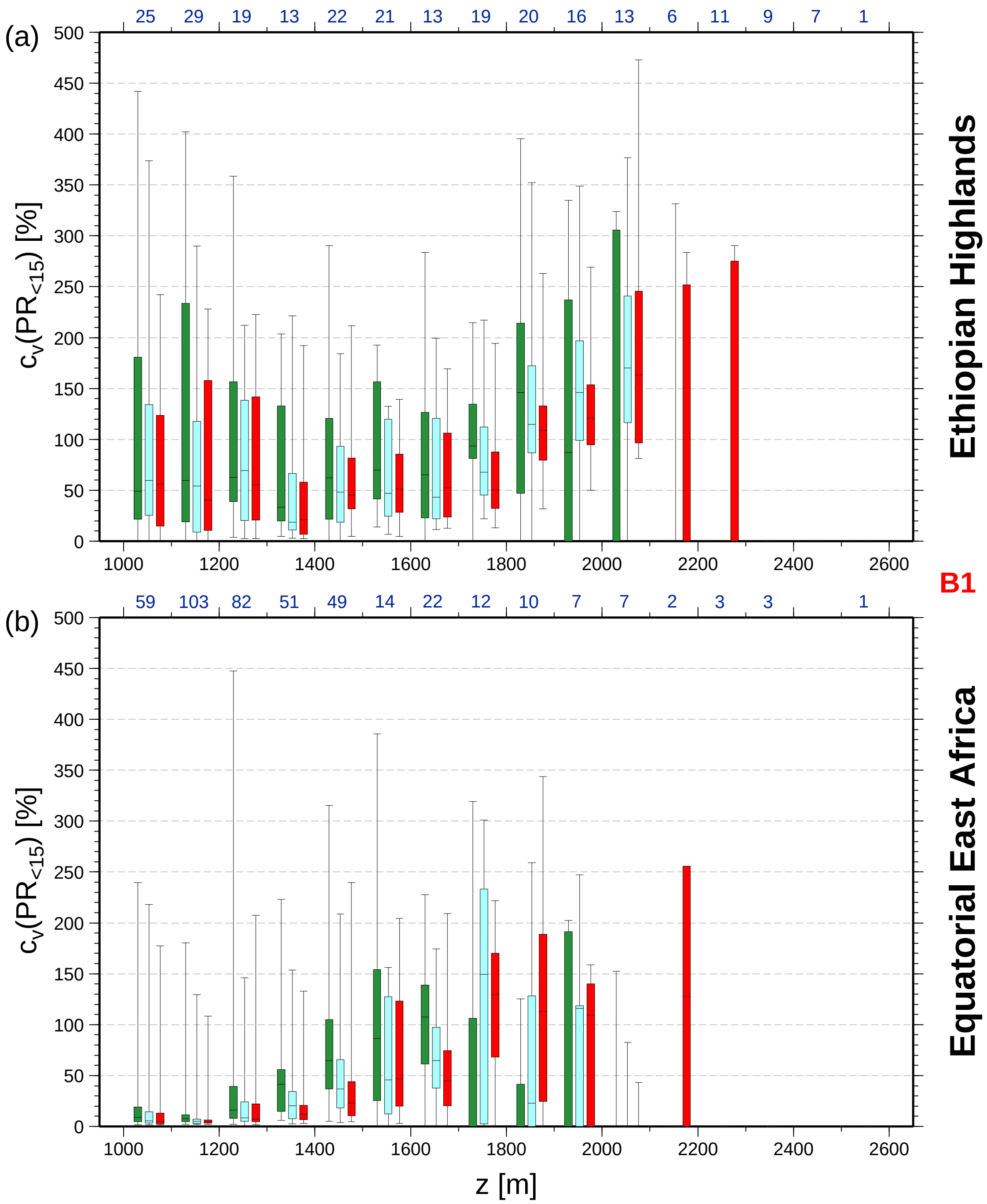

Supplemental Material, Figure 12: Same as Supplemental Material, Figure 7 but for the B1 scenario.

References

Bekessy A, Molineaux L, Storey Y. 1976. Estimation of incidence and recovery rates of *Plasmodium falciparum* parasitaemia, from longitudinal data. Bull World Health Org 54: 685–693.

Ermert V. 2010. Risk assessment with regard to the occurrence of malaria in Africa under the influence of observed and projected climate change. PhD thesis, University of Cologne, Cologne, Germany. Available: http://kups.ub.uni-koeln.de/volltexte/2010/3109/ [accessed 02 March 2011].

Ermert V, Fink AH, Jones AE, Morse AP. 2011a. A new version of the Liverpool Malaria Model. I. Refining the parameter settings and mathematical formulation of basic processes based on a literature review. Malar J, 10:35.

Ermert V, Fink AH, Jones AE, Morse AP. 2011b. A new version of the Liverpool Malaria Model. II. Calibration and validation for West Africa. Malar J, 10:62.

Faye O, Gaye O, Fontenille D, Hebrard G, Konaté L, Sy N et al. 1995. La sécheresse et la baisse du paludisme dans les Niayes du Sénégal. Cahiers Santé 5:299–305.

Fontenille D, Lochouarn L, Diatta M, Sokhna C, Dia I, Diagne N, et al. 1997. Four years' entomological study of the transmission of seasonal malaria in Senegal and the bionomics of *Anopheles gambiae* and *A. arabiensis*. Trans R Soc Trop Med Hyg 91:647–652.

Hay SI, Rogers DJ, Toomer JF, Snow RW. 2000. Annual *Plasmodium falciparum* entomological inoculation rates (EIR) across Africa: literature survey, internet access and review. Trans R Soc Trop Med Hyg 94:113–127.

Hay SI, Guerra CA, Gething PW, Patil AP, Tatem AJ, Noor AM et al. 2009. A world malaria map: *Plasmodium falciparum* endemicity in 2007. PLoS Medicine 6:e1000048.

Helsel DR, Hirsch RM. 2002. Statistical methods in water resources. Book 4, US Geological Survey, techniques of water-resources investigations, 510 pages. Available: http://water.usgs.gov/pubs/twri/twri4a3/ [accessed 02 March 2011].

Smith DL, Dushoff J, Snow RW, Hay SI. 2005. The entomological inoculation rate and *Plasmodium falciparum* infection in African children. Nature 438:492–495.
